# Factors Associated with Short and Long Term Mobility and HIV Risk of Women Living in Fishing Communities Around Lake Victoria in Kenya, Tanzania, and Uganda: A Cross Sectional Survey

**DOI:** 10.1007/s10461-022-03824-0

**Published:** 2022-09-10

**Authors:** Ubaldo M. Bahemuka, Paul Okimat, Emily L. Webb, Janet Seeley, Ali Ssetaala, Brenda Okech, Bertha Oketch, Freddie M. Kibengo, Elialilia Okello, Zachary Kwena, Monica O. Kuteesa, Matt A. Price, Pontiano Kaleebu, Heiner Grosskurth, Pat Fast, LVCHR

**Affiliations:** 1 Medical Research Council/Uganda Virus Research Institute Uganda Research Unit & London School of Hygiene and Tropical Medicine (MRC/UVRI & LSHTM) Uganda Research Unit, P.O. Box 49, Entebbe, Uganda; 2 London School of Hygiene and Tropical Medicine, London, UK; 3 UVRI-IAVI HIV Vaccine Program, Entebbe, Uganda; 4 Kenya Medical Research Institute, KEMRI, Kisumu, Kenya; 5 Mwanza Intervention Trials Unit, Mwanza, Tanzania; 6 International AIDS Vaccine Initiative, New York, USA; 7 University of California, San Francisco, San Francisco, USA

**Keywords:** Mobility, Factors, Women, Fishing communities, Lake Victoria

## Abstract

Mobility is linked to negative HIV care continuum outcomes. We sought to understand factors associated with short and long term mobility among women in fishing communities in Kenya, Tanzania, and Uganda. From 2018 through 2019 we conducted a cross-sectional survey of women aged 15 years and above, randomly selected from a census of six fishing villages, around Lake Victoria. Data collected included: demographics, risky sexual behaviour on the most recent trip, and travel behaviour in the previous 4 months. Mobility was recorded as any overnight trip outside the participant’s village. A two-level multinomial logistic regression model was used to determine the associated factors. A total of 901 participants were enrolled, of whom 645 (71.6%) reported travelling (53.4%; short and 18.2% long term trips). Five factors were associated with long term travel: age, travel purpose, frequency of travel, sexual behaviour while travelling, and destination. Trips made by women aged 46–75 years were less likely to be long term. Long term trips were more common if the trip was to visit, rather than to trade, and more common for women who reported one or two trips rather than three or more trips. Women who made long term trips were more likely to engage in unprotected sex while on a trip. Women who travelled to a regional town/district or another town/district were more likely to take long term trips. The factors associated with travel duration among women living in fishing communities could inform planning of future health care interventions in these communities.

**Supplementary Information:**

The online version contains supplementary material available at 10.1007/s10461-022-03824-0.

## Introduction

Mobility is known to increase risk for HIV infection by linking geographically separate epidemics, intensifying transmission by diffusing behavioural norms across social networks and enabling higher-risk sexual behaviour [1–8]. Mobility is also a potential barrier to HIV care and treatment for mobile individuals living with HIV as it affects entry into care and limits engagement and retention [9–11]. Similarly, high mobility complicates the development and testing of static HIV prevention interventions by limiting participation of mobile individuals [7].

A direct link between mobility and the risk of acquiring HIV has been documented in recent studies in Africa. Olawore et al. [12] demonstrated a direct link between migration and the risk of HIV acquisition for both women (1.75, 95% CI 1.33–2.33) and men (IRR 1.74, 1.12–2.71) compared to their non-migrant counterparts in Rakai District, southern Uganda.

Similarly, Dzomba et al. [13] found that high migration intensity was associated with an increased HIV acquisition risk among women when compared with low migration intensity (HR 2.88, 95% CI 1.56–5.53) in a recent surveillance cohort study in KwaZulu-Natal, South Africa. In the same population, another study by Dobra et al. [14] measured the space–time dimensions of human mobility in relationship to the risk of HIV acquisition and found a 50% increase in risk of acquiring HIV infection for mobility distances of 40 km and above. Kwena et al. [15] studied the link between mobility and sexual risk behaviour, and found a higher risk of sexual partner concurrency among both mobile men and women, but the risk of concurrency associated with mobility was stronger in women (aRR 2.0, 95% CI 1.1–3.7) than men (aRR 1.5, 95% CI 1.0–2.2). In addition, a previous study conducted among fisherfolk populations on Lake Victoria, found that females were more likely to be lost to follow-up from longitudinal studies than males [16]. However, this study was conducted with participants attending follow up visits at an inland fixed-based study site rather than a fishing community-based model.

We have been working with communities around Lake Victoria since 2009, studying issues related to HIV, including risk and health care access [17]. The mobility of the women in these populations has previously shown that they are involved in circular movements occurring between the fishing communities, urban fish markets and their rural hinterlands over long and short distances and periods of time, thus placing them beyond their boundaries of social norms and health care [18]. Qualitative data collection methods have been used in previous studies; a quantitative method study can provide further insight into predictors of duration, frequency, and patterns of mobility. In order to fill a gap in our understanding of the mobility duration characteristics of women in fishing community populations around Lake Victoria (Uganda, Tanzania and Kenya), we conducted a cross sectional survey in mainland and island fishing villages on Lake Victoria to understand the factors associated with short and long term mobility in this study population. We also considered the implications of mobility for the design and implementation of future health care interventions.

## Methods

### Study Design, Setting and Population

**Fig. 1:**
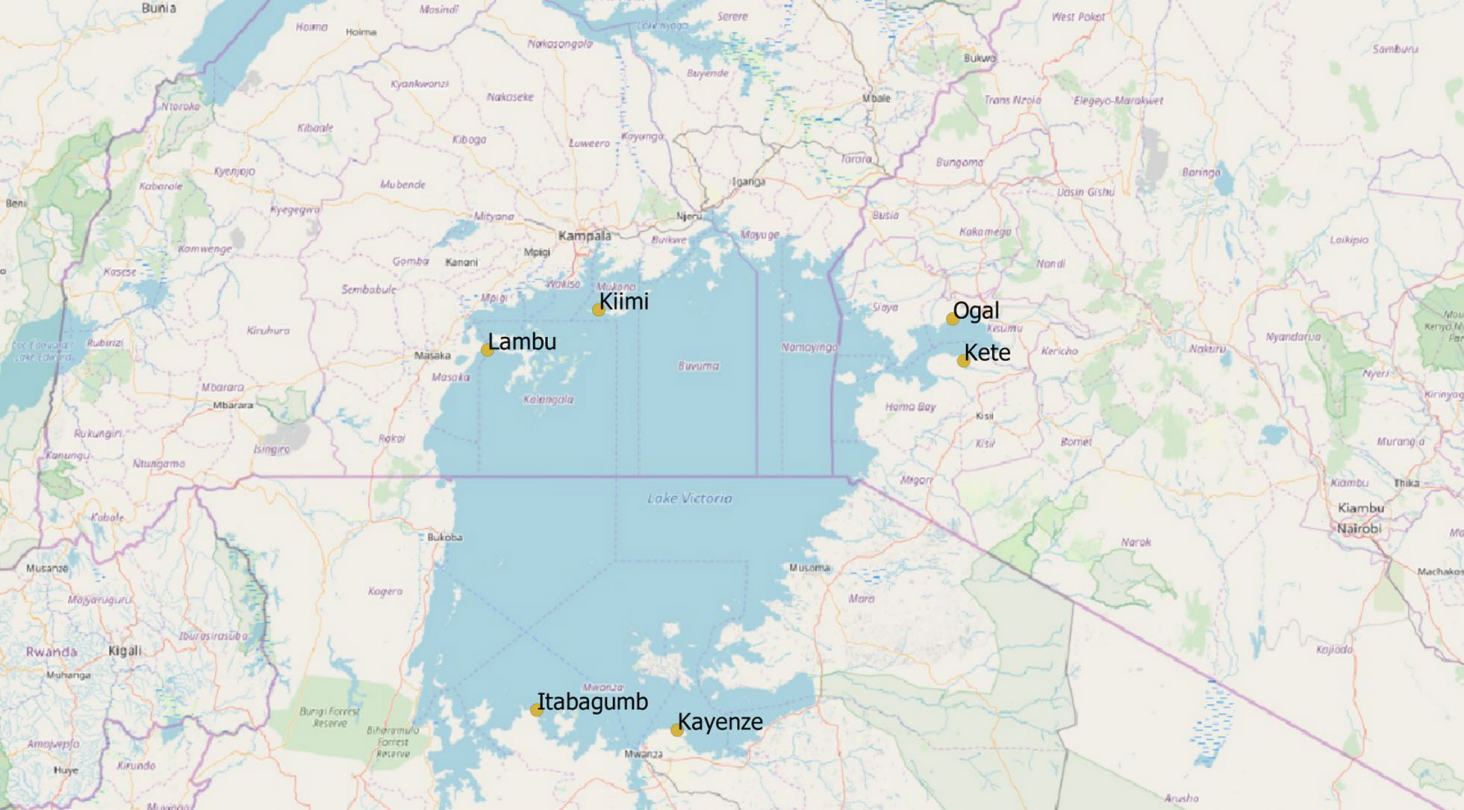
Map of Lake Victoria showing the participating fishing villages

The study protocol was jointly developed by investigators from the partners of the Lake Victoria Consortium for Health Research (LVCHR) [19]. The study described in this report is part of an on-going mixed methods (cross-sectional surveys and qualitative) multistage study, investigating several aspects of women’s mobility in fishing communities around Lake Victoria in Uganda, Tanzania, and Kenya. The study was conducted in Kete and Ogal (Kenya); Kayenze and Itabagumb (Tanzania); and Kiimi and Lambu (Uganda) (see Fig. 1). Each of the fishing communities was selected for having an estimated population of approximately 1000 residents composed of boat owners, fishermen, fish traders, shop owners living in proximity to subsistence farmers, and with HIV prevalence ranging between 15 and 34% among the members of the fishing communities, based on previous surveys in these populations [20, 21]. The women in these populations are commonly involved in activities such as bar work, food vending, sex work, and the fish trade.

### Sample Size and Sampling Procedure

Work began with community outreach and stakeholder engagement, including contacting area chiefs and assistant chiefs, the Beach Management Unit (BMU) officials (chief custodians of fish-landing sites), and community members through meetings involving the general community. Through this engagement, we introduced the study and the study team members, to gain local support and community buy-in. With the guidance of the community leaders, we mapped out community boundaries and generated a list of all dwellings (either residences or workplaces where people also stay) with women 15 years or older in each community. It was from this list that the sample of the women to participate was generated. The study statisticians generated random lists of approximately 600 women from each participating site. Thereafter, a smaller random sub-sample of 150 women was selected from each community to take part in the questionnaire survey, giving a final study sample size of approximately 900.

### Data Collection

Between January and June 2019, trained teams of male and female research assistants (RAs) sought informed consent from the randomly-selected potential participants. Upon consenting, the RAs conducted face-to-face, one-on-one structured interviews in a private location and in the participant’s language of choice, which was either English or local languages of Dholuo (Kenya), Kiswahili (Tanzania) and Luganda (Uganda). The interview included information on socio-demographic characteristics, travel patterns, related factors and HIV risk behaviours while on a trip. This interview lasted approximately 10 min including time for participants to recall the events from the last 4 months. The data were double checked by a peer reviewer before being uploaded onto the local servers at each participating site. The map showing the participating sites (Fig. 1) was developed using QGIS version 3.18.1, a free open source geographic information system.

### Statistical Analysis

The analysis was conducted using Stata software version 15. Age was summarised using median and interquartile ranges since it was not normally distributed, and categorical data were summarised using frequencies and percentages. We asked women about any overnight trips away from their village that they had taken in the past 4 months, and we then asked about mobility patterns in terms of mobility duration (nights away from village), and destination of the latest trip, and travel frequency (number of trips in past 4 months). We assessed factors associated with mobility duration, trichotomizing trips into one night away, two to six nights away, and seven or more nights away. Factors included for the analysis were sociodemographic [marital status, religious affiliation, age, highest level of education attained, number of biological children, occupation, and ethnicity (tribe)], travel-related behavioural characteristics (purpose of travel, travel destination, sexual activity, and alcohol consumption during trip), and the number of trips within 4 months preceding the interview. There was no special rationale for choosing 4 months’ recall, but we considered it was an adequate time-span to allow for participants to accurately recall the events and be able to capture as much data as possible. We employed χ^2^ statistics, and Fisher’s exact test, to test whether these factors varied across the three study countries and by the outcome of mobility duration. A two-level multinomial logistic regression was then used to determine the factors associated [estimated as ‘adjusted Prevalence Ratios’ (aPR)] with mobility duration (≤ a night, < a week, and ≥ a week). Clustering was adjusted for at country level. A two-level multinomial logistic regression model was found to suit the hierarchical nature of the data as compared to a three-level model based on both Akaike and Bayesian information criteria. A multilevel multinomial logit model is a mixed Generalized Linear Model with both linear predictors and a multinomial logit link [22, 23]. Our final model includes factors that remained significant at P < 0.05 or were implicated as confounders with other factors in the model.

$${{\varvec{\eta}}}_{{\varvec{i}}{\varvec{j}}}^{\boldsymbol{ }\left({\varvec{w}}\right)}={\boldsymbol{\alpha }}^{\left({\varvec{w}}\right)}+{{{\varvec{\beta}}}^{\left({\varvec{w}}\right)}}^{\boldsymbol{^{\prime}}}{{\varvec{x}}}_{{\varvec{i}}{\varvec{j}}}+{{\varvec{\xi}}\boldsymbol{ }}_{{\varvec{j}}}^{\boldsymbol{ }\left({\varvec{w}}\right)}+{{\varvec{\delta}}}_{{\varvec{i}}{\varvec{j}}}^{\left({\varvec{w}}\right)}$$

$$P({Y}_{ij}=w|{x}_{ij},{{\varvec{\xi}}}_{j},{{\varvec{\delta}}}_{ij})=\frac{\mathrm{exp}\{{{\varvec{\eta}}}_{{\varvec{i}}{\varvec{j}}}^{\boldsymbol{ }\left({\varvec{w}}\right)}\}}{1+{\sum }_{l=2}^{W}\mathrm{exp}\{{{\varvec{\eta}}}_{{\varvec{i}}{\varvec{j}}}^{\boldsymbol{ }\left({\varvec{l}}\right)}\}}$$

Y_ij_ is the outcome variable with a multinomial distribution (conditional on the random effects). Where, *w* = 1, 2, ..., *W* represents the response category (duration spent away from home), *j* = 1, 2, ..., *J* represents the cluster (country) and *i* = 1, 2,..., *nj* represents the subject (woman) of the *j*th cluster. ***ξ***_*j*_ and ***δ***_*ij*_ are vectors of random errors representing unobserved heterogeneity at cluster and subject level, respectively, taking on the following distribution assumptions:

- Errors at different levels are independent,
- $${{\varvec{\xi}}}_{{\varvec{j}}}^{\boldsymbol{^{\prime}}\boldsymbol{ }}={\left({\xi }_{{\varvec{j}}}^{\left(2\right)},\dots ,{\xi }_{{\varvec{j}}}^{\left({\varvec{W}}\right)}\right)}^{\boldsymbol{^{\prime}}}iid \,N(0,{\sum }_{{\varvec{\xi}}});$$

$$\varvec{\delta }^{\varvec{\prime}}_{\varvec{j}} = \left( {\delta _{{\varvec{ij}}}^{{\left( {\mathbf{2}} \right)}} , \ldots ,\delta _{{\varvec{ij}}}^{{\left( \varvec{W} \right)}} } \right)^{\varvec{\prime}} iid~N\left( {0,\Sigma _{\delta } } \right).$$

For multinomial models, the exponents of the coefficients are interpreted as Relative Risk Ratios (RRR) [24], however, since this study was conducted using a cross-sectional study design, we refer to them as Prevalence Ratios (PR). Our final model includes factors that remained significant at P < 0.05 or were implicated as confounders with other factors in the model.

### Ethical Considerations

Ethical approvals were granted by the Uganda Virus Research Institute (UVRI) Research Ethics Committee and the Uganda National Council for Science and Technology (UVRI REC#605 and SS#4470, Uganda); Kenya Medical Research Institute (KEMRI) Scientific and Ethics Review Unit (SERU#3593, Kenya); and National Health Research Ethics Committee (NatHREC & NIMR#2654, Tanzania). Study procedures were only conducted after obtaining written informed consent from a participant. Because there are many emancipated minors living in these communities, the study team requested and received a waiver for the requirement of parental consent from the ethical review committees to enrol individuals starting at the age of 15 years of age. Study information was shared by the study team initially in groups and later in one to one sessions. In cases where participants had a medical concern, they were referred by the study team staff for care. Each participant was reimbursed the equivalent of 5 USD in local currency for their time.

## Results

Between March 2018 and June 2019, a total of 901 female participants took part in the survey. The median age and interquartile ranges of the participants were as follows: overall 32 years (25, 40); Kenya 35 years (27, 48); Tanzania 29 years (23, 36); and 31 years (26, 38) in Uganda. Overall, most of the enrolled participants were either married or cohabiting (71.0%), with the majority with three or more children (62.4%) and having studied up to primary level (63.3%). The most commonly reported occupation was trading (23.6%), none self-reported being a sex worker for their occupation. Approximately 20.9% were unemployed or described themselves as ‘housewives’. There were significant differences across the three countries and sites for the majority of the variables, including marital status, numbers of children, and education levels (Table 1).

**Table 1:** Socio demographic characteristics of 901 female participants in six fishing communities on Lake Victoria shores in Kenya, Tanzania, and Uganda

Variable	Overall	Kenya	Tanzania	Uganda	Statistic	P value
Total enrolled	901	301	300	300		
Age in years (%)						
15–24	200 (22.2)	41 (13.6)	96 (32.0)	63 (21.0)	χ^2^(4) = 80.6	< 0.001
25–45	567 (62.9)	176 (58.5)	176 (58.7)	215 (71.7)		
46–75	134 (14.9)	84 (27.9)	28 (9.3)	22 (7.3)		
Marital status (%)						
Married/Cohabiting	640 (71.0)	225 (74.8)	199 (66.3)	216 (72.0)	χ^2^(6) = 143.1	< 0.001
Separated	105 (11.7)	5 (1.7)	42 (14.0)	58 (19.3)		
Widowed	90 (10.0)	65 (21.6)	14 (4.7)	11 (3.7)		
Single/never married	66 (7.3)	6 (2.0)	45 (15.0)	15 (5.0)		
Number of biological children (%)						
0	73 (8.1)	12 (4.0)	41 (13.7)	20 (6.7)	χ^2^(4) = 36.7	< 0.001
1–2	266 (29.5)	70 (23.6)	105 (35.0)	91 (30.3)		
≥ 3	562 (62.8)	219 (72.8)	154 (51.3)	189 (63.0)		
Religious affiliation (%)						
Catholic	269 (29.9)	33 (11.0)	96 (32.0)	140 (46.7)	χ^2^(6) = 250.9	< 0.001
Moslem	106 (11.8)	6 (2.0)	36 (12.0)	64 (21.3)		
Pentecostal	133 (14.8)	30 (10.0)	58 (19.3)	45 (15.0)		
Other	393 (43.6)	232 (77.1)	110 (36.7)	51 (17.0)		
Highest education level attained (%)						
No formal education	144 (15.9)	16 (5.3)	100 (33.3)	28 (9.3)	χ^2^(6) = 150.9	< 0.001
Primary	570 (63.6)	223 (74.9)	174 (58.0)	173 (57.7)		
Secondary	161 (17.9)	46 (15.8)	23 (7.7)	92 (30.7)		
Tertiary	26 (2.9)	16 (5.3)	3 (1.0)	7 (2.3)		
Occupation (%)						
Trader	213 (23.6)	76 (25.3)	85 (28.3)	52 (17.3)	χ^2^(10) = 173.8	< 0.001
Farming	127 (14.1)	54 (17.9)	71 (23.7)	2 (0.7)		
Fishing or related activities	150 (16.7)	27 (9.0)	32 (10.7)	91 (30.3)		
Housewife/Unemployed	188 (20.9)	87 (28.9)	62 (20.7)	39 (13.0)		
Other	223 (24.8)	57 (18.9)	50 (16.7)	116 (8.7)		

Other reported religious affiliations include Anglican, Lutheran, 7th day Adventist and other independent churches/sects (e.g. Vosh, Roho, True church)

Other reported occupations/activities include operating bars or restaurants, hair dressing, operating mobile money shops, teaching, health worker

*IQR* interquartile range

### Mobility, Mobility Patterns and Risk Behaviour While Travelling

A total of 645 (71.6%) of the participants reported travelling out of the fishing community in the 4 months prior to the interview; 213 (70.8%), 205 (68.3%), and 227 (75.7%) in Kenya, Tanzania, and Uganda, respectively (Table 2). Out of the 645 that reported travelling, 267 (41.4%), 126 (19.5%), and 240 (37.2%) reported making one, two, or ≥ 3 trips out of the community, respectively. Frequency of travel, and length and destination of most recent trip also varied between countries (Table 2).

**Table 2:** Travel patterns and HIV risk related behavioural characteristics of female participants in six fishing communities on Lake Victoria shores in Kenya, Tanzania, and Uganda

Variable	Overall	Kenya	Tanzania	Uganda	Statistic	P value
Frequency of travel in the past 4 months (%)						
None	256 (28.4)	88 (29.2)	95 (31.7)	73 (24.3)	χ^2^(6) = 18.6	0.005
Once	267 (29.6)	98 (32.6)	93 (31.0)	76 (25.3)		
Twice	126 (14.0)	30 (10.0)	39 (13.0)	57 (19.0)		
More than twice	240 (26.6)	84 (27.9)	66 (22.0)	90 (30.0)		
Do not remember	12 (1.3)	1 (0.3)	7 (2.3)	4 (1.3)		
Time spent away from the fishing community in the most recent trip (%)						
No mobility reported	256 (28.4)	88 (29.2)	95 (31.7)	73 (24.3)	χ^2^(10) = 51.6	< 0.001
One night or less	276 (30.6)	112 (37.2)	89 (29.7)	75 (25.0)		
2–6 nights	205 (22.8)	68 (22.6)	49 (16.3)	88 (29.3)		
7–30 nights	127 (14.1)	24 (8.0)	55 (18.3)	48 (16.0)		
31–90 nights	30 (3.3)	3 (1.0)	11 (3.7)	16 (5.3)		
> 90 nights	7 (0.8)	6 (2.0)	1 (0.3)	0 (0.0)		
Travel destination during the most recent trip (%)						
Another fishing community	100 (15.5)	34 (16.0)	38 (19.0)	28 (12.3)	χ^2^(8) = 87.8	< 0.001
Another inland village	67 (10.4)	22 (10.3)	37 (18.1)	8 (3.5)		
Regional town/district	176 (27.3)	59 (27.7)	75 (36.6)	42 (18.5)		
Another town/district	286 (44.4)	98 (46.1)	54 (26.4)	134 (59.3)		
Elsewhere	16 (2.5)	0 (0.0)	1 (0.5)	15 (6.6)		
Sexual activity during the most recent trip (%)						
Yes	50 (7.8)	15 (7.3)	15 (7.3)	20 (8.8)	χ^2^(2) = 0.6	0.756
No	595 (92.3)	198 (93.0)	190 (92.7)	207 (91.2)		
Alcohol consumption during the most recent trip (%)						
Yes	62 (9.6)	12 (5.6)	20 (9.8)	30 (13.2)	χ^2^(2) = 7.3	0.026
No	583 (90.4)	201 (94.4)	185 (90.2)	197 (86.8)		

### Factors Associated with Mobility Duration

In univariable analysis of correlates of mobility duration, significant associations were observed for age, marital status, occupation, travel destination, sexual activity while on trip, and travel purpose (Tables 3, 4).

**Table 3:** Unadjusted associations between socio demographic characteristics, and duration spent out of the community among females who participated in the survey

Variable (category)	No trip	One night	> One night, < One week	≥ One week	Statistic	P value
Age (n, %)						
15–24	63 (24.6)	54 (19.6)	33 (16.1)	50 (30.5)	χ^2^(6) = 19.1	< 0.01
25–45	155 (60.5)	170 (61.6)	142 (69.3)	100 (61.0)		
46–75	38 (14.8)	52 (18.8)	30 (14.6)	14 (8.5)		
Marital status						
Married/Cohabiting	175 (68.4)	195 (70.7)	153 (74.6)	117 (71.3)	χ^2^(9) = 19.6	0.02
Separated	26 (10.2)	32 (11.6)	28 (13.7)	19 (11.6)		
Widowed	29 (11.3)	36 (13.0)	15 (7.3)	10 (6.1)		
Single/never married	26 (10.2)	13 (4.7)	9 (4.4)	18 (11.0)		
Number of biological children						
0	22 (8.6)	21 (7.6)	13 (6.3)	17 (10.4)	χ^2^(6) = 4.8	0.57
1–2	80 (31.3)	76 (27.5)	57 (27.8)	53 (32.3)		
≥ 3	154 (60.2)	179 (64.9)	135 (65.9	94 (57.3)		
Religious affiliation						
Catholic	76 (29.7)	76 (27.5)	68 (33.2)	49 (29.9)	χ^2^(9) = 12.3	0.20
Moslem	22 (8.6)	31 (11.2)	24 (11.7)	29 (17.7)		
Pentecostal	38 (14.8)	40 (14.5)	34 (16.6)	21 (12.8)		
Other	120 (46.9)	129 (46.7)	79 (18.5)	65 (39.6)		
Highest education level attained						
No formal education	50 (19.5)	40 (14.5)	28 (13.7)	26(15.9)	χ^2^(9) = 11.6	0.24
Primary	154 (60.2)	188 (68.1)	133 (64.9)	95 (57.9)		
Secondary	46 (118.0)	38 (13.8)	39 (19.0)	38 (23.2)		
Tertiary	6 (2.3)	10 (3.6)	5 (2.4)	5 (3.0)		
Occupation						
Trader	62 (24.2)	28 (12.1)	16 (9.2)	26 (14.9)	χ^2^(12) = 28.3	0.01
Farming	45 (17.6)	23 (9.9)	14 (8.0)	11 (8.0)		
Fishing or related activities	37 (14.5)	115 (49.6)	94 (54.0)	78 (56.5)		
Housewife/Unemployed	64 (25.0)	34 (14.7)	26 (14.9)	13 (9.4)		
Other	48 (18.8)	32 (13.8)	24 (13.8)	23 (16.7)

**Table 4:** Unadjusted associations between travel related characteristics, and duration spent out of the community among females who participated in the survey

Variable (category)	One night	> One night, < One week	≥ One week	Statistic	P value
Travel destination					
Another fishing community	59 (21.4)	19 (9.3)	22 (13.4)	χ^2^(8) = 58.7	< 0.01
Another inland village	48 (17.4)	10 (4.9)	9 (5.5)		
Regional town/district	74 (26.8)	49 (23.9)	53 (32.3)		
Another town/district	93 (33.7)	119 (58.0)	74 (45.1)		
Elsewhere	2 (0.7)	8 (3.9)	6 (3.7)		
Frequency of trips					
Once	82 (29.7)	91 (44.4)	94 (57.3)	χ^2^(6) = 49.0	< 0.01
Twice	48 (17.4)	44 (21.5)	34 (20.7)		
More than twice	140 (50.7)	65 (31.7)	35 (21.3)		
Do not remember	6 (2.2)	5 (2.4)	1 (0.6)		
Sexual activity while on trip					
Yes (used a condom)	4 (1.4)	1 (0.5)	9 (5.5)	Fisher’s exact test	< 0.01
Yes (did not use a condom)	5 (1.8)	16 (7.8)	15 (9.1)		
No	267 (96.7)	188 (91.7)	140 (85.4)		
Alcohol consumption while on trip					
Yes	24 (44.4)	17 (32.7)	21 (51.2)	χ^2^(2) = 2.60	0.27
No	30 (55.6)	35 (67.3)	20 (48.8)		
Purpose for travel					
Trading	44 (16.0)	8 (3.9)	10 (6.1)	χ^2^(10) = 107.2	< 0.01
Visit	44 (16.0)	60 (29.3)	73 (44.5)		
Going home	3 (1.1)	14 (6.8)	8 (4.9)		
Seeking healthcare	63 (22.9)	13 (6.3)	17 (10.4)		
> 1 reason	116 (42.2)	98 (47.8)	43 (26.2)		
Other reasons	5 (1.8)	12 (5.9)	13 (1.9)		
Suspicion about partner having sex with another person					
Definitely is	64 (29.8)	48 (30.6)	49 (39.2)	χ^2^(8) = 12.9	0.12
Probably is	36 (16.7)	22 (14.0)	13 (10.4)		
Probably is not	7 (3.3)	11 (7.0)	3 (2.4)		
Definitely is not	39 (18.1)	25 (15.9)	13 (10.4)		
Not sure	69 (32.1)	51 (32.5)	47 (37.6)		

### Age

In the multivariable analysis (Table 5), travel duration varied with age group, with longer trips decreasing as age increased. Compared to those aged 15–24 years, a trip made by participants aged 46–75 years was less likely to last a week or more, than to be a single-night trip, aPR 0.41 (95% CI 0.18, 0.91), Z-statistic = − 2.19, P = 0.029.

**Table 5:** Adjusted associations between participant characteristics (socio-demographic & travel-related behavioural), and trip duration among females who participated in the survey (n = 645)

Variable	aPR (95% CI)	Z-statistic	P	aPR (95% C I)	Z-statistic	P
	> One night, < One week vs			≥ One week vs		
	≤ One night (base outcome)			≤ One night (base outcome)		
Age (years)						
15–24	(1 ref)			(1 ref)		
25–45	1.14 (0.66, 1.96)	0.47	0.636	0.66 (0.39, 1.13)	− 1.50	0.132
46–75	0.87 (0.44, 1.74)	− 0.39	0.697	0.41 (0.18, 0.91)	− 2.19	0.029
Travel purpose						
Trading	(1 ref)			(1 ref)		
Visit (friend/family/partner)	5.23 (2.14, 12.81)	3.62	< 0.001	4.89 (1.98, 12.10)	3.44	0.001
Going home	15.16 (3.35, 68.61)	3.53	< 0.001	9.03 (1.81, 45.10)	2.68	0.007
Seeking health care	1.13 (0.41, 3.11)	0.23	0.819	1.06 (0.39, 2.90)	0.11	0.909
More than one reason	9.00 (2.22, 36.59)	3.07	0.002	10.08 (2.42, 41.99)	3.17	0.002
Other	3.52 (1.49, 8.21)	2.88	0.004	1.37 (0.56, 3.40)	0.69	0.492
Travel frequency						
More than twice	(1 ref)			(1 ref)		
Once	2.08 (1.31, 3.30)	3.10	0.002	4.33 (2.54, 7.37)	5.39	< 0.001
Twice	1.61 (0.93, 2.78)	1.69	0.092	2.37 (1.26, 4.45)	2.68	0.007
Do not remember	2.18 (0.51, 9.28)	1.05	0.294	0.49 (0.05, 4.92)	− 0.61	0.543
Sexual activity while on trip						
No	(1 ref)			(1 ref)		
Yes (used a condom)	0.62 (0.06, 6.26)	− 0.41	0.685	4.44 (1.05, 18.88)	2.02	0.043
Yes (did not use a condom)	5.24 (1.76, 15.63)	2.97	0.003	5.86 (1.90, 18.06)	3.08	0.002
Travel destination						
Another fishing community	(1 ref)			(1 ref)		
Another village	0.89 (0.36, 2.22)	− 0.25	0.802	0.73 (0.28, 1.92)	− 0.64	0.521
Regional town/district	2.27 (1.14, 4.51)	2.33	0.020	2.25 (1.12, 4.52)	2.27	0.023
Another town/district	3.52 (1.87, 6.64)	3.89	< 0.001	2.06 (1.06, 4.01)	2.13	0.034
Elsewhere	6.02 (0.98, 36.78	1.94	0.052	3.80 (0.56, 25.95)	1.36	0.173

*PR* prevalence ratio

### Travel Purpose

In the multivariable analysis we found that there were differences in mobility duration patterns by travel purpose. For instance, a trip to visit, rather than to trade, was more likely to last for up to a week than to be a single-night trip, and even more likely to last for more than a week than to be a single night (aPR 5.23, 95% CI 2.14–12.81, Z-statistic = 3.62, P < 0.001 and 4.89, 95% CI 1.98–12.10, Z-statistic = 3.44, P = 0.001, respectively). A trip home rather than to trade was more likely to last for up to a week than to be a single-night trip, and more likely to last for more than a week than a single night (aPR 15.16, 95% CI 3.35–68.61, Z-statistic = 3.53, P < 0.001 and 9.03, 95% CI 1.81–45.10, Z-statistic = 2.68, P = 0.007, respectively). Making a trip for more than one reason rather than to trade (alone) was also more likely to last for up to a week and even more likely to last for more than a week (compared to up to a night); aPR 9.00, 95% CI 2.22–36.59, Z-statistic = 3.07, P = 0.002 and 10.08, 95% CI 0.56–3.40, Z-statistic = 3.17, P = 0.002, respectively. Taking a trip for other reasons (not categorised) rather than to trade was more likely to last more than a night (but less than a week) rather than for a night: aPR 3.52, 95% CI 1.49–8.21, Z-statistic = 2.88, P = 0.004.

### Travel Frequency

In the adjusted analysis, travel frequency was inversely associated with the length of the most recent trip. Specifically, a participant who made one trip was more likely than one who made more than two trips to make a trip lasting up to a week and even more likely to make a trip lasting for more than a week (rather than up to a night): aPR 2.08, 95% CI 1.31–3.30, Z-statistic = 3.10, P = 0.002 and 4.33, 95% CI 2.54–7.37, Z-statistic = 5.39, P < 0.001, respectively. In addition, a participant who made two trips was more likely than one who made more than two trips, to make a trip that lasted a week or more rather than up to a night (aPR 2.37, 95% CI 1.26–4.45, Z-statistic = 2.68, P = 0.007).

### Sexual Behaviour

In the adjusted analysis, there was a general pattern of participants being more likely to engage in sexual activity during longer trips. Participants who engaged in protected sex while on a trip were more likely than those who did not engage in sex at all to make a trip lasting more than a week (compared to a night): aPR 4.44, 95% CI 1.05–18.88, Z-statistic = 2.02, P = 0.043. Participants who engaged in unprotected sex while on a trip were more likely than those who did not engage in sex to make a trip lasting up to a week and similarly more likely to make a trip lasting for more than a week (compared to a night): aPR 5.24, 95% CI 1.76–15.63, Z-statistic = 2.97, P = 0.003 and 5.86, 95% CI 1.90–18.06, Z-statistic = 3.08, P = 0.002, respectively.

### Travel Destination

In the multivariable analysis, travel duration varied with destination. Compared to those who travelled to another fishing village, those who travelled to a regional town (or district) were more likely to make a trip lasting up to a week or more (compared to a single-night trip): aPR 2.27, 95% CI 1.14–4.51, Z-statistic = 2.33, P = 0.020 and 2.25, 95% CI 1.12–4.52, Z-statistic = 2.27, P = 0.023, respectively. Similarly, compared to those who travelled to another fishing village, those who travelled to another town (or district) were more likely to make a trip lasting up to a week or more (compared to a single-night trip): aPR 3.52, 95% CI 1.87–6.64, Z-statistic = 3.89, P < 0.001 and 2.06, 95% CI 1.06–4.01, Z-statistic = 2.13, P = 0.034, respectively.

## Discussion

Our findings show that there is high mobility among the women from Lake Victoria fishing communities in Kenya, Tanzania and Uganda, as previously noted [18, 25, 26]. Mobility among women in fishing communities has been linked to HIV acquisition and may be viewed as a time when women may engage in high risk sexual behaviour, away from the gendered checks and balances within their communities [15, 25, 27]. Most of the mobility was to locations outside fishing communities. This finding may be due to the fact that most residents of these Lake Victoria fishing communities are dependent on fishing related activities, hence, mobility among female members of the fishing communities may have been due to the changes in fish seasons requiring movement to agricultural communities to boost income during periods when the fish catch is low [28]. Women’s travels outside fishing communities could also be for trade reasons, to sell fish to better markets outside the fishing communities, as has been previously noted [18]. Mobility for sex work could also have contributed to women’s movements to locations outside fishing communities as previously documented [18], although we could not ascertain the extent of such movement, probably due to social desirability bias.

Our findings also indicate that older age was associated with fewer longer periods of travel, probably due to societal, trade and family commitments that come with ageing, which eventually limit older fishing community members’ travel. This finding is contrary to previous work where older women travelled for longer periods for trade [18]. Movements outside fishing communities to regional towns or districts were associated with longer travel periods, perhaps because such mobility is often for trading, looking for better markets. That said, longer travel periods were associated with many other reasons rather than for trade only. Since most fishing communities are hard to reach, women could have opted to move to accomplish multiple goals during travel.

Some participants engaged in risky sexual activity during travel, a finding that would require further investigation. Participants who did have sexual activity during travel were less likely to have used condoms, contrary to some of the previous work indicating that women who had sex during travel were using condoms [15]. Condom-less sexual activities during travel were likely to be more among participants who had moved for longer periods. This is consistent with previous work and may be due to the limited social scrutiny during longer travel periods as sexual behaviour is often not controlled by societal relationships or family ties due to their absence during travel [18, 29]. Equally such travel could be to meet long-term partners with women travelling to their marital homes and hence sex was condom-less. Women who travelled for longer periods might have perceived themselves to be at lower risk of sexually transmitted infections, including HIV. Our previous work indicates that women who engaged in sex work, bar or restaurant work had shorter travel periods [18], such women are often at high risk for infections and may perceive themselves at increased risk of HIV acquisition.

Based on our findings planning and implementation of future longitudinal studies would require an initial period to be able to reduce follow up in the active phases of the trial. The observed mobility and associated factors highlight the need for investment in sophisticated technology and methods that can be suited to maintaining contact with participants as they go about their livelihoods. For instance, the younger women, who are the majority, would require a form of communication to be able to reach them wherever they are.

This study was not without limitations. Firstly, selection of study communities was not representative of all fishing communities; therefore, results may not be generalizable to all fishing communities on Lake Victoria. Secondly, participants might have been affected by social desirably bias and under report sexual behaviours during travel. We minimised this through the use of experienced interviewers, who are not members of these study communities, but were well known and trusted by the communities. Thirdly, our study might have been affected by recall bias, with participants having challenges recalling past events related to their mobility. We minimized this through using a relatively short recall period of 4 months.

Despite the above limitations, this study is valuable in providing additional information on factors associated with mobility of women to inform the planning and design of future HIV prevention interventions with women in these communities. The findings of this study may inform future work on the adoption of artificial intelligence technologies used to increase retention in clinical trials conducted in these populations [30].

## Conclusion

There was high mobility among the majority of women in this study, often to locations outside their primary fishing communities with some travels involving high risk sexual activities. The understanding of the association between: age, travel; purpose, frequency and destination and travel duration, is useful for planning future healthcare interventions in the women of these communities. Careful considerations on retention need to be made regarding the involvement of these women in long-term health care interventions such as clinical research.

## Supplementary Information

Below is the link to the electronic supplementary material.

Supplementary file1 (DOCX 142 kb)

## Data Availability

Data will be available on a link provided.

## Code Availability

A link to access the code will be provided.
