# Supplementary material for: Factors Associated with Short and Long Term Mobility and HIV Risk of Women Living in Fishing Communities Around Lake Victoria in Kenya, Tanzania, and Uganda: A Cross Sectional Survey
Source: AIDS Behav. 2022 Sep 10;27(3):880–90. doi: 10.1007/s10461-022-03824-0 (PMC9944640; doi:10.1007/s10461-022-03824-0)
Supplement: Supplementary file 1 — Supplementary file1 (DOCX 142 kb) [file 10461_2022_3824_MOESM1_ESM.docx]

**Study Title: Mobility Patterns and feasibility of Tracking Women at High Risk of HIV in the Fishing Communities in Kenya, Tanzania and Uganda**

LVCHR study Code: Mob-001

Country code : Kenya (254), Tanzania (255), Uganda (256) |__|__|__|[CTCODE]

Fishing community: Lambu (), Kiimi (54), Igombe ( ), Kijiweni (), Kenya? |__|__|[FCCODE]

Start time: |__|__|: |__|__| (24 hour clock) [STATTIMEM]

End time: |__|__|: |__|__| (24 hour clock) [ENDTIMEM]

Data collector # |__|__|__|[INTERNUM]

Date of data collection ____/____/201___ [DATE]

DD/MM / YYYY

| **Section 1: Demographic Data** | | | | | | |
| --- | --- | --- | --- | --- | --- | --- |
| **In this section, we are going to ask you some general questions about yourself, like your age, education, current marital status and work experience.** Mukitundu kino, tugenda kukubuuza ebibuuzo ebikukwatako, okugeza; emyakagyo, obuyigirize, ebyobufumbo, n’emirimu. | | | | | | |
|  | |  | |  |  | |
| 1.1 | | Gender of participant  **Ekikula ky’omuntu** | | 1=Female  2=Male | SEX | |
| 1.2 | | How old are you? (Indicate age in years)  **Olina emyaka emeka?** | |  | AGE0 | |
| 1.3 | | Date of birth  **Wazaalibwa ddi?** | | \|D D\|M MM\|Y Y Y Y\|  Put 99 for unknown DD,999 for unknown MMM and 9999 for unknown YYYY but ask for the age as they know it and record it. | BIRTHDATE | |
| 1.4 | | Place of birth  **Wazaalibwa wa?** | | 1= Current fishing community  2=Other fishing village  3=Regional district  4=Other town/District  5=Elsewhere (specify) ……………………… | BIRTHPLACE  OTHERBIRTH | |
| 1.5 | | What is your current marital status?  **Oli mufumbo?** | | 1=Married/Cohabiting  2=Separated  3=Widowed  4= Single/Never Married | MARITAL | |
| 1.6 | | How many biological children do you have?  **Olina abaana bameka ?** | | 1=None  2=1-2  3=3+  9= Prefer not to answer | CHILDBIO | |
| 1.7 | | What is your tribe?  **Oli wa ggwanga ki?** | | 1=Muganda  2=Munyankole/Mukiga  3=Munyoro/Mutooro  4=Mufumbira  5=Other Ugandan ethnic group  6=Other East African ethnic group  7=Other ethnic group from Africa/elsewhere  (If 5, 6 or 7, specify)……………………….. | TRIBE  TRIBEOTHT | |
| 1.8 | | What is your religious affiliation?  **Osoma ddiini ki?** | | 1=Catholic  2=Lutheran  3=Anglican  4=Moslem  5=Pentecostal  6=Seventh Day Adventist  7=Other (Specify)…….......... | RELIGION  OTHERRELIG | |
| 1.9 | | What is your highest Education level?  **Wakomawa mu kusoma?** | | 1=Primary  2=S.1 - 4  3=S.5 - 6  4=Tertiary  5=No formal Education | LEVELED | |
| 1.10 | | What is your main occupation?  **Okola mulimo ki (omukulu)?**  (*Interviewer: do NOT prompt any of the following! Record spontaneous response).* | | 1=Trader  2=Bar /restaurant worker  3=Sex worker (self-identified)  4=Farming  5=Fishing or related activities  6=Housewife / unemployed  7=Other (specify)……………………… | OCCUPATION  OTHEROCCUP | |
| **Section 2: Mobility patterns (Recent mobility)** | | | | | | |
| **In this section, we are going to ask about travels that you made outside this community during the past 4 months. Please try to recall the different movements you made in the last 4 months and exactly what happened and as I mentioned earlier, all the information you give would only be used for aggregate purposes and we will ensure all the confidentiality that it requires.**  Mu kitundu kino, tugenda kukubuuza ku kutambulakwo ng’ofulumamu okuva mu kitundu muno mu bbanga ery’emyezi ena egiyise. Nkusaba ogezeeko okujjukira emirundi gy’ofulumyemu mu kitundu mu bbanga ery’emyezi ena egiyise na kiki ddala ekyaliwo. Era nga bwanakugambye mu kusooka, amawulire gonna gotuwa gajja kukozesebwa mu kugerageranya era tujja kukakasa nti gakuumibwa nga ga kyama. | | | | | | |
| 2.1 | How long have you lived in this community?  **Omaze bbanga ki mu kitundu Kin0?** | | 1= Born in this community Q2.4  2= Less than one year  3= More than one year  8= Don’t remember  **Skip to 2.4 if code is 1** | | | LIVECOMTY |
| 2.2 | Where did you live before coming to this community?  **Wava wa okujja mu kitundu kino?** | | 1=Never lived outside this community  2=Other fishing community  3=Non fishing community  -  4=Elsewhere (specify)………………………………. | | | LIVEBFR  LIVELSE |
| 2.3 | Why did you move to this …..community (mention the community)?  **Lwaki wajja mu kitundu kino?** | | 1= Work  2= Came to live with partner/family  3= Born here  4= Other (specify) …………………………. | | | WHYMOVE  RESNMOV |
| 2.4 | Have you moved out of this community in the last 4 months?  **Ofulumyeko okuva mu kitundu muno mu bbanga ery’emyezi ena egiyise?**  If 2 skip to section 3.0 | | 1=Yes 󠅄  2=No Q3.0 | | | TRAVELOUT |
| 2.5 | How many times have you moved in and out of this community in the past 4 months?  **Mirundi emeka gy’ofulumye okuva mu kitundu kino mu bbanga ery’emyezi ena egiyise?** | | 1=Once  2=Twice  3=More than Twice  8=Don’t remember | | | TIMESTRAVEL |
|  | ***Interviewer:*** *if participant moved more than once then fill Q 2.5 for the latest trip; and Q 2.6 for the previous. If more than 2 trips, only record the last two trips.* | | | | | |
| 2.5.0 | **Latest travel** (omulundi gwasembyeyo okufuluma okuva mu kitindu kino) | |  | | |  |
| 2.5.1 | When was the last time you moved out of this community?  **Wasembayo ddi okufuluma mu kitundu kino?** | | 1=Less than a week ago  2=Over a week ago  3=More than a month ago  4=More than 4 months ago  8=Don’t remember | | | LASTTRAVEL |
| 2.5.2 | Where did you go?  **Walaga wa?** | | 1=Another fishing community  2=Another village (not fishing)  3=Regional town/district  4=Another town/ district  5=Elsewhere (Specify) ……………………….. | | | GOWHERE  GOWHERESP |
| 2.5.3 | What was the main purpose of this travel?  **Nsongaki enkulu eyali ekutambuzizza/ekututte?** | | *Please do NOT prompt! Record spontaneous response (tick all that apply)* | | | TRAVELPURP |
|  | 1=Trading | |  | | | TRADEOT |
|  | 2= Bar work | |  | | | BARWORKOT |
|  | 3=Sex work | |  | | | SEXWORKOT |
|  | 4=Visit friends/family/partner | |  | | | VISITFROT |
|  | 5=Going home | |  | | |  |
|  | 6=Seeking health care  7=Other (specify) | | *…………………………………………………* | | | OTRAVTSP |
| 2.5.4 | How long were you away from this community?  **Wamalayo bbanga ki?** | | 1=One day  2=Less than a week  3=Over a week  4=Over a month  5=Over 3 months  8=Don’t remember | | | AWAYCOM |
| 2.5.5 | Did you engage in any sexual activity during this travel?  **Wegattako mu by’omukwano ku mulundi guno gwewatambula?**  (If 2 Skip to 2.6 **[previous travel])** | | 1=Yes  2=No Q2.6  8=Don’t Remember  4=Prefer not to answer | | | SEXAWAY |
| 2.5.6 | What relationship do/did you have with this sexual partner/s?  **Walina/olina nkolaganaki n’lwaono /abagalwa bano gwe/bewegatta naye/nabo?** | | 1=Husband  2=Boyfriend  3=Casual sexual partner  4=Other (please specify): _____________  Multiple responses apply code 7 for blank | | | RELPATAWAY  RELPATSP |
| 2.5.7 | Did you use a condom the last time you had sex with this partner?  **Mwakozesa akapiira ku mulundi gwemwasembayo okwegatta mu kikolwa eky’ekyama?** | | 1=Yes  2=No  8=Don’t Remember  9=Prefer not to answer  **If didn’t use on some partners , code 2** | | | CONDAWAY |
| 2.5.8 | Did you receive anything (e.g. gift, fish, favors or the right to buy your fish) in exchange for sex?  **Yakuwayo ekintu kyonna (okugeza; ekirabo, eby’enyanja, okuyambibwa oba okukuguza/okugula eby’enyanjabyo olw’okwegatta naye mu kikolwa eky’ekyama?)** | | 1=Yes  2=No  8=Don’t Remember  9=Prefer not to answer | | | GIFTRECEIVE |
| 2.5.9 | Do you still expect to have sex with this partner(s) when you travel again?  **Osuubira okuddamu okwegatta mu kikolwa eky’omukwano n’omwagalwa ono/abagalwa bano omulundi omulala lw’onotambula?** | | 1=Yes  2=No  8=Don’t Remember  9=Prefer not to answer | | | SEXEXPT |
| 2.6.0 | **Previous travel** (omulundi ogw’emabega gwe watambula) | |  | | |  |
| 2.6.1 | When was the previous time you moved out of this community?  **Ddi lwewafulumamu okuva mu kitundu kino omulundi ogwakulembera?** | | 1=Less than a week ago  2=Over a week ago  3=More than a month ago  4=More than 4 months ago  8=Don’t remember | | | PREVTRAVEL |
| 2.6.2 | Where did you go?  **Walaga wa?** | | 1= Another fishing community  2=Another village (not fishing)  3=Regional town  4=Another town/district  5=Elsewhere (specify) ……………………… | | | WHERGO2  WHERGO2SP |
| 2.6.3 | What was the main purpose of this travel?  **Nsonga ki enkulu eyali ekututte?** | | *Please do NOT prompt! Record spontaneous response (tick all that apply)* | | | TRAVELPURP2 |
|  | 1=Trading | |  | | |  |
|  | 2=Bar work | |  | | |  |
|  | 3=Sex work | |  | | |  |
|  | 4=Visit friends/family/partner | |  | | |  |
|  | 5=Going home | |  | | |  |
|  | 6=Seeking health care | |  | | |  |
|  | 7=Other (specify) | | *…………………………………………………* | | | TRAVELOT2 |
| 2.6.4 | How long were you away from this community?  **Wamalayo bbanga ki?** | | 1=Less than a week  2=Over a week  3=Over a month  4=Over 3 months  8=Don’t remember | | | TIMEAWAY |
| 2.6.5 | Did you engage in any sexual activity during this travel?  **Wegattako mu kikolwa eky’ekyama ku mulundi guno gwewatambula?**  If 2, Skip to 3.0 | | 1=Yes  2=No  3=Don’t Remember  4=Prefer not to answer | | | SEXAWAY2 |
| 2.6.6 | What relationship do/did you have with this sexual partner?  **Walina/olina nkolaganaki n’omwagalwa ono gwe wegatta naye mu kikolwa eky’ekyama?** | | 1=Wife/husband  2=Boyfriend/girlfriend  3=Casual sexual partner  4=Other (please specify): _____________ | | | RELATPART2  OTHERRELA2 |
| 2.6.7 | Did you use a condom the last time you had sex with this partner?  **Mwakozesa akapiira ku mulundi gwemwasembayo okwegatta mu kikolwa eky’ekyama?** | | 1=Yes  2=No  8=Don’t Remember  9=Prefer not to answer | | | CONDPART2 |
| 2.6.8 | Did you receive anything (e.g. gift, fish, favors or the right to buy your fish) in exchange for sex?  **Yakuwayo ekintu kyonna (okugeza; ekirabo, eby’enyanja, okuyambibwa oba okukuguza/okugula eby’enyanjabyo olw’okwegatta naye mu kikolwa eky’ekyama?)** | | 1=Yes  2=No  8=Don’t Remember  9=Prefer not to answer | | | GIFTRECEIVE2 |
| 2.6.9 | Do you still expect to have sex with this partner(s) when you travel again?  Osubiira okuddamu okwegatta mu byómukwano n’omwagalwa/abagalwa bwóddamu okutambula? | | 1=Yes  2=No  9=Prefer not to answer | | | SEXEXPT2 |
| **Section 3: Mobility within the community and sex generated mobility** | | | | | | |
| **Now we are going to talk about the times you slept outside your home while in your community as well as the times you moved for sex during the past 4 months. As was the case in the previous section, please try to recall the different times you spent a night out of your home while in the community and say exactly what happened. You will also indicate when you travelled purposely for sex. As I mentioned earlier, all the information you give would only be used for aggregate purposes and we will ensure all the confidentiality that it requires.**  Kati tugenda kwogera ku mirundi gyotasuze wakawo naye ng’oli mu kitundu muno wamu n’emirundi gy’otambudde ng’ogenda kwegatta mu kikolwa eky’ekyama mu bbanga ery’emyezi ena egiyise. Era ng’ebitundu ebikulembedde, gezaako okujjukira emirundi egyenjawulo gy’otosuze waka wo naye ng’oli mu kitundu na kiki ddala ekyaliwo. Ojja kulaga ne lwewatambula ng’ogenda kwegatta mu kikolwa eky’ekyama. Nga bwenayogedde mu kusooka, amawulire gonna g’onoowa gakweyambisa mukugerageranya era gajja kukuumibwa mu kyama. | | | | | | |
| 3.1 | | Have you spent a night outside your home while in your community in the past 4 months?  **Osuzeeko wa bweru w’amakago naye ng’oli mu kitundu muno mu bbanga ery’emyezi ena egiyise?**  (If No skip to 3.7) | | 1=Yes  2=No | TRAVELIN | |
| 3.2 | | How many times did you spend a night outside your home while in this community in the past 4 months?  **Mirundi emeka gy’otaasula waka wo naye ng’oli mu kitundu mu bbanga ery’emyezi ena egiyise?** | | 1=Once  2=Twice  3=More than twice  8=Don’t remember  9=Prefer not to answer | TIMESIN | |
| 3.3 | | Where did you spend the night?  **Wasula wa?** | | 1=Friend’s house  2=Partner’s house  3=Entertainment place  4=Elsewhere (Specify) ……………………… | WHEREIN  WHEREINSP | |
| 3.4 | | What was the main purpose of your movement within this community?  **Nsonga ki enkulu eyali ekututte?** | | *Please do NOT prompt! Record spontaneous response (tick all that apply)* | TRAVELPURP3 | |
|  | | 1=Trading | |  | TRADEIN | |
|  | | 2=Bar work | |  | BARWORKIN | |
|  | | 3=Sex work | |  | SEXWORKIN | |
|  | | 4=Visit friends/family/partner | |  | VISITFRIN | |
|  | | 5= Going home | |  | GOHMIN | |
|  | | 6=Entertainment | |  | ENTIN | |
|  | | 7=Seeking health care  8=Other (specify) | | *…………………………………………………* | OTHERTRAVIN | |
| 3.5 | | Did you engage in any sexual activity during this movement within  **Wegattako mu byómukwano ku mulundi guno gwewatambula ngóli mu kitundu kyo?**  If 2, Skip to 4.0 | | 1=Yes  2=No Q.4.0  8=Don’t Remember  9=Prefer not to answer | SEXIN | |
| 3.6 | | What relationship do/did you have with this sexual partner/s?  **Walina/olina nkolaganaki n’lwaono /abagalwa bano gwe/bewegatta naye/nabo?** | | 1=husband  2= Boyfriend  3= Casual sexual partner/s  4= Other (please specify): ____________  Multiple responses apply, code 7 in blank | RELPARTIN  OTRELPATIN | |
| 3.7 | | Did you make any movements within this community purposely for sex?  **Wavakomumakaago mu kitundu kino ng’ogenda kwegatta mu by’omukwano?***  If no, skip to 4.0 | | 1=Yes  2=No Q.4.0  9=Prefer not to say | SEXTRAVEL | |
| 3.8 | | Where did you go?  **Walaga wa?** | | 1=Friend’s house  2=Partner’s house  3=Entertainment place  4=Elsewhere (Specify) ……………………… | SEXGO | |
| 3.9 | | What relationship do/did you have with the partner/s you had sex with?  **Olina/walina nkolagana ya ngeri ki n’omwagalwa gwegatta naye muby’ómukwano?** | | 1= husband  2=Boyfriend  3=Casual sexual partner  4=Other (please specify): _____________ | RELATIONPART | |
| 3.10 | | How many times did you move within this community purposely for sex during the past 4 months?  **Mirundi emeka gyewatambula mu kitundu kino ng’ogenda kwegatta mu by’**  **omukwano mu bbanga ery’emyezi ena egiyise?** | | 1=Never  2=Once  3=More than once  8=Don’t remember  9=Prefer not to answer | SEXTRAVTMS | |

| **Section 4: Sexual Behaviour and Partners’ mobility** | | | |
| --- | --- | --- | --- |
| **In this section, we are going to ask you about your sexual behaviour and the travels that your partner made outside this community during the past 4 months. Please try to remember exactly what happened and answer the following questions**  Mu kitundu kino, tugenda kukubuuza ebikwatagana n’okwegatta mu kikolwa eky’ekyama wamu n’engendo omwagalwawo zeyatambula mu bbanga ery’emyezi ena egiyise. Nkusaba ogezeko okujjukira ekyo ekyaliwo era oddemu ebibuuzo bino. | | | |
| 4.1 | In the past 4 months, have you had sex with any Partner?  **Mu bbanga ery’emyezi ena egiyise, wegasseko n’omwagalwa yenna mu kikolwa eky’ekyama?**  If 2 Skip to 5.1 | 1= Yes  2= No  8= Don’t Remember  9= Prefer not to answer | SEX4 |
| 4.2 | What relationship do/did you have with this sexual partner/s?  **Walina/olina nkolaganaki n’lwaono /abagalwa bano gwe/bewegatta naye/nabo??** | 1= Husband  2= Boyfriend  3= Casual sexual partner  4= Other (please specify): _____________ | RELPATSEX  OTRELSEX |
| 4.3 | What is your partner’s main occupation? *(for women with regular partners)*  **Omwagalwawo akola mulimu ki omukulu?** (*eri abakyala abalina abagalwa abenkalakkalira)* | 1=Fishing or related activities  2=Trader  3=Farming  7=Other (Specify): ______________ | OCUPART  OTOCUPAT |
| 4.4 | Has your partner/s moved out of/into this community in the past 4 months?  **Omwagalwawo avuddeko/azzeko mu kitundu kino mu bbanga ery’emyezi ena egiyise?**  If 2 skip to 4.9 | 1=Yes  2=No Q.4.9  8=Don’t Remember | PARTMOVE |
| 4.5 | How many times has your partner/s moved out of/into this community in the past 4 months?  **Mirundi emeka omwagalwawo gyavudde/gy’azze** **mu kitundu kino mu bbanga ery’emyezi ena egiyise?** | 1=Once  2=Twice  3=More than twice  8=Don’t remember | PARTOUT |
| 4.6 | When was the last time your partner/s travelled out of/into this community?  **Ddi omwagalwawo lwe yasembayo okuva/okujja mu kitundu kino?** | 1=Less than a week  2=Over a week back  3=Less than a month back  4=Over a month back  8=Don’t remember | LSTPARTMV |
| 4.7 | Where did your partner go/come from?  **Omwagalwawo yagenda/yava wa?** | 1=Another fishing community  2=Another village (not fishing)  3=Regional town/district  5=Another town/district  6=Elsewhere (specify) ……………………… | PRTWHER  OTPRTWHE |
| 4.8 | What was the purpose of your partner’s travel?  **Nsonga ki enkulu eyali etutte/ereese omwagalwawo?** | 1= Fishing or related activities  2= Trading  3= Visit friends or relatives  4= Other (specify)_____________  5= Don’t know  8= I don’t remember | PRTPURPS  OTPATPUR |
| 4.9 | How long was your partner away from/in this community?  **Omwagalwawo yamalayo/yamalawo bbanga ki?** | 1=Less than a week  2= Over a week  3=Over a month  4=Over 3 months  8=Don’t remember | LONGPART |
| 4.10 | How likely is it that your partner is having sex with another partner besides you?  **Olowooza omwagalwawo alina omwagalwa omulala gweyegatta naye mu by’omukwano?** | 1=Definitely is  2=Probably is  3=Probably is not  4=Definitely is not  5=Not sure  6=Prefer not to answer | PARTSEXOUT |
| 4.11 | Did you engage in any sexual activity while your regular partner was away?  **Wegattako mu byómukwano mu kiseera ng’omwagalwawo taliiwo?**  If 2, skip to 5.0 | 1=Yes  2=No Q.5.0  8= Don’t remember  9=Prefer not to answer | SEXPTAWAY |
| 4.12 | What was/is your relationship with this other partner?  **Walina/olina nkolagana ya ngeri ki n’omwagalwa on’omulala?** | 1=Wife/husband  2=Boyfriend/girlfriend  3=Casual sexual partner  4=Other (please specify): _____________ | RLTPTWAY  OTRLTPAW |
| 4.13 | Did you use condoms the last time you had sex with this other partner?  **Mwakozesa akapiira nga mwegatta mu byómukwano n’omwagalwa on’ómulala ku mulundi gwemwasembayo?** | 1=Yes  2= No  8= Don’t Remember  9= Prefer not to answer | CONPATWAY |
| 4.14 | How many sexual partners have you had sex with in the last 4 months?  **Bagalwa bameka bewegasse nabo mu muby’omukwano mu bbanga ery’emyezi ena egiyise?** | 1=Only one  2=More than one  3=None  8=Don’t remember  9=Prefer not to answer | SEXPART |
| 4.15 | How frequently did you use condoms during sex?  **Obupiira wabukozesanga kwenkanawa nga wegatta mu kikolwa eky’ekyama?** | 1=Never used condoms  2=Sometimes  3=All the time | CONUSEFREQ |
| **SECTION 5: ALCOHOL USE**  **Now I will ask you about any alcoholic drinks that you may have used during your movements outside this community. The reason we are asking is that we want to understand better what may put people at risk of illness or injury whilst they travel. The questions about alcohol drinks may be sensitive for some people. Remember that you are free not to respond to these questions if you do not want to.**  Kati ngenda kukubuuza ku by’okunywa ebitamiiza by’okozesezza ku mirundi gy’otambudde okuvaako mu kitundu muno. Ensonga lwaki tubuuza nti twagala okutegeera obulungi biki ebiyinza okuteeka abantu mu katyabaga k’endwadde oba okukosebwa nga batambudde. Ebibuuzo ebikwata ku mwenge abantu abamu biyinza okubayisa obubi. Jjukira nti oli wa ddembe obutaddamu bibuuzo bino singa oba toyagadde. | | | |
| 5.1 | Do you take alcohol?  **Onywa ku mwenge?** | 1=Yes  2=No | ALCOHOL |
| 5.2 | During your travels out of this community, did you take any drink with alcohol such as beer, wine or spirit  **Emirundi egyo gyewatambula okuvaako mukitundu muno, olina ekitamiiza kyonna kyewanywa okugeza bbiya, wayini oba waragi?** | 1=Yes  2=No  8=Don’t remember  9=Prefer not to answer | ALCOTRAVEL |
| 5.3 | What type of drink did/do you take?  **Kyakunywa ki kyewanywa?** | 1=Beer (commercially produced)  2=Beer (locally brewed)  3=wine (commercially produced)  4=Wine (locally brewed)  5=Spirit or other strong drink  6=Beer and/or wine and also spirit | ALCOTYPE |
| 5.34 | On a typical day, how many drinks do you take?  **Mu lunaku onywa omwenge gwenkanaki?** | 1=One drink  2=Two drinks  3=Three to five drinks  4=More than five drinks | ALCOQUANT |

Thank you for participating.

**Webale kwetaba mu kunonyereza.**
